# Development of a ferritin-based nanoparticle vaccine against the SARS-CoV-2 Omicron variant

**DOI:** 10.1038/s41392-022-01041-8

**Published:** 2022-06-01

**Authors:** Wanbo Tai, Benjie Chai, Shengyong Feng, Xinyu Zhuang, Jun Ma, Mujia Pang, Lin Pan, Zi Yang, Mingyao Tian, Gong Cheng

**Affiliations:** 1 Institute of Infectious Diseases, Shenzhen Bay Laboratory, Shenzhen, Guangdong China; 2 Tsinghua-Peking Joint Center for Life Sciences, School of Medicine, Tsinghua University, Beijing, China; 3 Changchun Veterinary Research Institute, Chinese Academy of Agricultural Sciences, Changchun, China

**Keywords:** Vaccines, Vaccines

**Dear Editor**,

The COVID-19 pandemic has had a devastating effect on global health, resulting in over 6.2 million deaths worldwide. Continuous emergence of adaptive mutations of SARS-CoV-2 alters its pathogenicity and transmissibility, and renders its resistance to current vaccines and antiviral drugs.^1^ A new variant named Omicron discovered initially in South Africa has recently been proposed as a variant of concern (VOC) by the World Health Organization, because of its high transmissibility and resistance to current vaccines and therapeutic antibodies.^2^ Therefore, development of vaccines against prevalent variants including Omicron is urgently needed for COVID-19 prevention.

A previous study developed a SARS-CoV-2 vaccine based on a virus-like nanoparticle (VLP) platform, in which sixty copies of a fusion protein including a receptor binding domain (RBD) with a lumazine synthase as the structural scaffold were self-assembled into a nanoparticle.^3^ Based on this framework, we further designed a self-assembling ferritin-based nanoparticle (FNP) vaccine against the SARS-CoV-2 Omicron variant. In this system, twenty-four copies of ferritin containing an N-terminal protein A tag form a structural scaffold (Fig. 1a). The RBD (residues 331aa-524aa) of the SARS-CoV-2 Omicron spike protein with an Fc tag in the C-terminus (Fc-RBD_Omicron_) served as an essential immunogen (Fig. 1a).^4^ The purified Fc-RBD_Omicron_ automatically assembled onto the nanoparticles by the Fc-protein A tag interaction (Fig. 1a). Based on this concept, the antigen of emerging SARS-CoV-2 variants can be assembled onto nanoparticles through a separating preparation and a subsequent Fc-Protein-A-tag-mediated conjugation. Of note, accumulating evidence indicate that the neutralizing potency elicited by a SARS-CoV-2 RBD dimer was much stronger than that by an RBD monomer.^5^ Therefore, this vaccine strategy may show advantage to stimulate the neutralizing immune responses than the previous design.

**Fig. 1:**
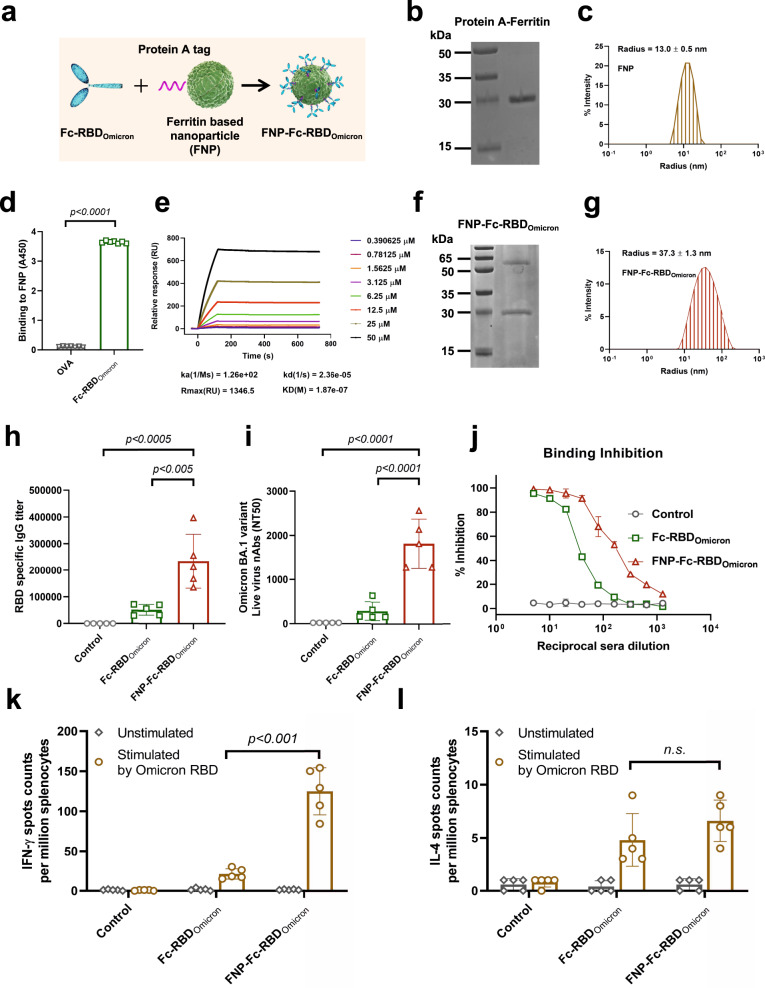
Development and characterization of the FNP-Fc-RBDOmicron vaccine against SARS-CoV-2 Omicron variant. **a** Schematic representation of a SARS-CoV-2 Omicron RBD with Fc tag (light green), a ferritin-based 24-meric nanoparticle with N-terminal protein A tag (green), and an FNP-Fc-RBD_Omicron_ complex. **b** The FNP complex was analyzed by SDS-PAGE. **c** Size distribution of the FNP complex was detected by DLS. **d**, **e** Interaction between Fc-RBD_Omicron_ and FNP was detected by ELISA and SPR. An equal amount of ovalbumin served as a control. The data are presented as mean ± S.E.M. Statistical significance was calculated via ordinary unpaired parametric *t* test. **f** The FNP-Fc-RBD_Omicron_ complex was analyzed by SDS-PAGE. **g** Size distribution of the FNP-Fc-RBD_Omicorn_ was detected by DLS. **h**, **i** Measurement of IgG and neutralizing antibodies induced in immunized mice. The mice were immunized via intramuscular (i.m.) prime and boost at 2 weeks (10 μg per mouse, *n* = 5). Sera at 14 days post-2nd immunization were detected for RBD_Omicron_-specific IgG antibodies by ELISA. The neutralizing antibodies were assessed by live SARS-CoV-2 Omicron BA.1 virus. The data are presented as mean ± S.E.M. (*n* = 5). Statistical significance was calculated via one-way ANOVA with multiple comparisons test. **j** Inhibition potency of immunized sera on SARS-CoV-2 RBD-hACE2 binding in hACE2/HEK293T cells. The inhibition potency was evaluated by flow cytometry. Inhibition percentage (%) was calculated by a relative fluorescence intensity. **k**, **l** Splenocytes were stimulated with the RBD protein of Omicron. The IFN-γ and IL-4 secretion condition in splenocytes were detected by an ELISpot assay. Data represented as mean ± S.E.M. (*n* = 5). Statistical significance was calculated via ordinary unpaired parametric *t* test

We expressed and purified the ferritin containing an N-terminal protein A tag in *Escherichia coli* (Supplementary Fig. S1a), and its purity was confirmed by SDS-PAGE (Fig. 1b). The characterization of the self-assembling nanoparticles was analyzed by negative-stain electron microscopy (EM) (Supplementary Fig. S1b) and dynamic light scattering (DLS) (Fig. 1c). The results indicated that the nanoparticles were spherical with a uniform diameter of 13.0 ± 0.5 nm. We next expressed the Fc-RBD_Omicron_ in the FreeStyle 293-F cells (Supplementary Fig. S2a, b). The binding affinity of Fc-RBD_Omicron_ for hACE2 was evaluated by both enzyme linked immunosorbent assay (ELISA) (Supplementary Fig. S3a) and flow cytometry (Supplementary Fig. S3b) with a dose-dependent manner. We next assembled the Fc-RBD_Omicron_ onto the 24-meric FNP by mixing these two components at a 24:1 molar ratio. The Fc-RBD_Omicron_ was capable of tightly interacting with the nanoparticles through the Fc-Protein A, measured by ELISA (Fig. 1d) and surface plasmon resonance technology (SPR) (Fig. 1e). The protein complex was co-eluted and co-purified by gel filtration chromatography, and further evaluated by SDS-PAGE (Fig. 1f). The protein complex was designated as the FNP-Fc-RBD_Omicron_ throughout this investigation. Furthermore, the FNP-Fc-RBD_Omicron_ complex was evaluated by DLS, which confirmed the diameter of FNP-Fc-RBD_Omicron_ being uniformly about 37.3 ± 1.3 nm (Fig. 1g). Altogether, we generated self-assembling ferritin-based nanoparticles to develop a vaccine against the SARS-CoV-2 Omicron variant.

We next evaluated the potency of the FNP-Fc-RBD_Omicron_ to induce immune responses against SARS-CoV-2. To this end, we immunized hACE2-transgenic mice with either FNP-Fc-RBD_Omicron_ or a sole Fc-RBD_Omicron_. The mice were further boosted with the same dose of immunogens at 2 weeks after the primary immunization. Mouse sera were collected on Day 14 after the second immunization and analyzed for antibody titers and potency to neutralize SARS-CoV-2. The SARS-CoV-2 Omicron RBD-specific IgG titer induced by FNP-Fc-RBD_Omicron_ was 4 times higher than that by Fc-RBD_Omicron_ (Fig. 1h). Subsequently, the neutralizing potency in the sera of immunized animals was assessed by authentic SARS-CoV-2 Omicron virus. The sera showed a higher neutralizing activity in the FNP-Fc-RBD_Omicron_ immunized mice than that of a sole Fc-RBD_Omicron_ immunization (Fig. 1i).

To substantiate the SARS-CoV-2-neutralizing mechanism of vaccine-induced antibodies, we examined the interactions between the SARS-CoV-2 RBD and hACE2 in the presence of the vaccinated mouse sera by flow cytometry. Although the binding of RBD_Omicron_ to hACE2/HKE293T cells was inhibited by either FNP-Fc-RBD_Omicron_ or Fc-RBD_Omicron_ serum effectively in a dose-dependent manner, the former was more potent (Fig. 1j and Supplementary Fig. S4). We examined whether the antibodies induced by FNP-Fc-RBD_Omicron_ immunization could interrupt the entry of HIV pseudotyped with spike of SARS-CoV-2 VOCs. Indeed, FNP-Fc-RBD_Omicron_ vaccinated sera effectively blocked the cellular entry of multiple SARS-CoV-2 VOCs (Supplementary Fig. S5). Nonetheless, the sera showed less neutralizing activity against other SARS-CoV-2 VOCs than that of Omicron, suggesting the diverse antigenicity between Omicron and other SARS-CoV-2 variants (Supplementary Fig. S5). To assess ability and duration of immune protection by FNP-Fc-RBD_Omicron_ vaccination, we measured the RBD specific IgG level and neutralizing activity in the sera of FNP-Fc-RBD_Omicron_ immunized animals on 21 and 42 days after the booster vaccination. The neutralizing potency in the sera maintained a high level on these 2 time points (Supplementary Fig. S6), indicating vaccination of FNP-Fc-RBD_Omicron_ elicited a prolonged immune protection in animals. We next assessed the cellular immune responses in the mice immunized by FNP-Fc-RBD_Omicron_. The splenocytes were isolated from either immunized or control mice at 45 days after the booster dose. Subsequently, the splenocytes were in vitro stimulated by the purified RBD proteins of Wildtype (Supplementary Fig. S7a, b), Delta (Supplementary Fig. S7c, d) and Omicron BA.1 (Fig. 1k, l) variants. The RBD specific spots numbers of IFN-γ-, but not IL-4-producing T cells, was significantly higher in the FNP-Fc-RBD_Omicron_ immunized animals than that in the Fc-RBD_Omicron_ group, indicating a Th1 biased cellular immune response. Of note, the Wildtype and Delta RBD antigens also induces antigen-specific cellular immune responses in the FNP-Fc-RBD_Omicron_ immunized animals (Supplementary Fig. S7). Overall, immunization of FNP-Fc-RBD_Omicron_ stimulated antigen-specific humoral and cellular responses to multiple SARS-CoV-2 VOCs, thus indicating that the Omicron RBD-based vaccine may serve as a booster against COVID-19.

Overall, these results demonstrate that immunization of a self-assembling ferritin-based nanoparticle vaccine offers a robust humoral immune response against Omicron variant. Herein, a pseudovirus-based neutralization assay confirmed that vaccination with FNP-Fc-RBD_Omicron_ could provide an effective neutralizing potency against both Omicron BA.1 and BA.2 variant infection. This study offers a great potential for the quick response of the emerging SARS-CoV-2 variants and affords versatility to develop vaccines against other emerging and reemerging coronaviruses in future.

## Supplementary information

Supplementary Materials

## Data Availability

All data and materials that support the findings of this study are available from the corresponding author upon reasonable request.
