# Supplementary material for: Development of a ferritin-based nanoparticle vaccine against the SARS-CoV-2 Omicron variant
Source: Signal Transduct Target Ther. 2022 Jun 1;7:173. doi: 10.1038/s41392-022-01041-8 (PMC9157036; doi:10.1038/s41392-022-01041-8)
Supplement: Supplementary file 1 — Supplementary Materials [file 41392_2022_1041_MOESM1_ESM.docx]

Supplementary Materials for

Development of a ferritin-based nanoparticle vaccine against the SARS-CoV-2 Omicron variant

Wanbo Tai^1,2,#^, Benjie Chai^2,#^, Shengyong Feng^2,#^, Xinyu Zhuang^3,#^, Jun Ma^1^, Mujia Pang^1^, Lin Pan^1,2^, Zi Yang^2^, Mingyao Tian^3,*^, Gong Cheng^2,*^

**Affiliations:**

^1^ Institute of Infectious Diseases, Shenzhen Bay Laboratory, Shenzhen, Guangdong, China.

^2^ Tsinghua-Peking Joint Center for Life Sciences, School of Medicine, Tsinghua University, Beijing, China.

^3^ Changchun Veterinary Research Institute, Chinese Academy of Agricultural Sciences, Changchun, China.

^#^These authors contributed equally to this work.

*Corresponding to gongcheng@mail.tsinghua.edu.cn (G.C.) and klwklw@126.com (M.T.).

**This PDF file includes:**

Materials and Methods

Figures. S1 to S7

References

Materials and Methods

**Cell lines and plasmids**

HEK293T cells (human embryonic kidney cells) and Vero cells (CCL-81) was obtained from the American Type Culture Collection (ATCC) and cultured in Dulbecco’s modified Eagle medium (DMEM, supplemented with 10% fetal bovine serum and 100 units/mL penicillin-streptomycin). hACE2/HEK293T cells were kindly given by professor Qiang Ding from Tsinghua University and cultured with the same condition as HEK293T cells. The FreeStyle 293-F Cells were purchased from Gibco and cultured in the FreeStyle 293 Expression Medium (Gibco) (Supplemented with 100 units/mL penicillin and 100 μg/mL streptomycin). BL21 (DE3) *E. coli* cells were obtained from TransGen Biotech and cultured in Luria-Bertani (LB) medium containing 50 μg/mL Kanamycin.

The genes encoding SARS-CoV-2 Omicron variant spike (GISAID accession ID: EPI_ISL_6640916), ferritin (NCBI Reference Sequence: WP_000949190.1), and domain B of protein A (residues 212aa–270aa) from *S. aureus* (NCBI Reference Sequence: WP_190282922.1) were all optimized and synthesized by GenScript. The SARS-CoV-2 Omicron variant RBD (residues 331aa-524aa) was subcloned into pFuse-hIgG1-Fc2 vector (InvivoGen). Ferritin was subcloned into pET-28a c (+) vector with an N-terminal domain B of protein A and His×8-tag. Plasmids expressing the spike proteins of SARS-CoV-2 wild type and various variants pcDNA3.1-WT-S (Wildtype strain, GenBank accession number: NC_045512.2) pcDNA3.1-B.1.1.7-S (Alpha variant, GISAID accession ID: EPI_ISL_601443), pcDNA3.1-B.1.351-S (Beta variant, GISAID accession ID: EPI_ISL_700428), pcDNA3.1-P.1-S (Gamma variant, GISAID accession ID: EPI_ISL_792680), pcDNA3.1-B.1.617-S (Delta variant, GISAID accession ID: EPI_ISL_2461258), pcDNA3.1-B.1.1.529-BA.1-S (Omicron variant, GISAID accession ID: EPI_ISL_6640916) and pcDNA3.1-B.1.1.529-BA.2-S (Omicron variant, GenBank accession number: UJL09565.1) pNL4-3.luc.RE (the luciferase reporter-expressing HIV-1 backbone) were constructed previously and maintained in our laboratory.

**Protein expression and purification**

SARS-CoV-2 RBD protein was expressed from the FreeStyle 293-F cells. Briefly, the Fc-tagged RBD (Fc-RBD_Omicron_) was collected from the cell culture medium, purified using Protein A column and Superdex 200 Increase 10/300 GL gel filtration chromatography. His-tagged ferritin-based nanoparticle (FNP) was expressed from BL21 (DE3) *E. coli* cells. Protein expression was induced using IPTG (isopropyl-beta-D-thiogalactoside) at a final concentration of 1 mM and purified using Ni-NTA column and Superdex 200 Increase 10/300 GL gel filtration chromatography. The purified FNP and Fc-RBD_Omicron_ proteins were co-incubated (molar ratio is 1:24) at room temperature for 1 hour and subsequently the formed complex was purified using gel filtration chromatography. The diameters of FNP and FNP-Fc-RBD_Omicron_ were characterized with a dynamic light scatter (DLS, Wyatt Technology), and the purified proteins were analyzed by SDS-PAGE.

**Surface plasmon resonance (SPR) analysis**

The binding kinetics of Fc-RBD_Omicron_ with FNP were analyzed by SPR (Biacore 8K, GE Healthcare). Specifically, the FNP were transferred into HBST buffer (20 mM HEPES (pH 7.4), 150 mM NaCl, and 0.005% (v/v) Tween 20) and immobilized on the CM5 chip (Cytiva). Then, serially diluted Fc-RBD_Omicron_ samples (from 50 μM to 0.390625 μM) flowed over the chip in PBST buffer. BSA protein was selected as negative control. Binding affinities were measured using a BIAcore 8K (GE Healthcare) at 25℃ in the multi-cycle mode. Binding kinetics were analyzed with Biacore^TM^ Insight software (GE healthcare) using a 1:1 Langmuir binding model.

### Negative staining analysis

Negative-staining electron microscopy procedures were conducted as previously described.^1^ Briefly, the purified FNP sample of 5 μl with a final concentration of 0.15 mg/ml in PBS was loaded onto a freshly glow-discharged carbon coated grid (230 mesh, Beijing Zhongjingkeyi). After incubating for 1 min, excess sample was blotted, and the grid was stained with 5 μl 2% (w/v) uranyl acetate solution for 1 min. Excess solution was blotted and grids were dried at room temperature. Images were acquired using a Tecnai Spirit (FEI) operated at 120 kV and 4 K × 4 K Ultrascan CCD camera at 98,000× magnification at the Institute of Biophysics, Chinese Academy of Sciences.

**Mouse immunization**

Four-week-old hACE2 transgenic mice were immunized with FNP-Fc-RBD_Omicron_ protein (10 μg/mouse), Fc-RBD_Omicron_ protein (10 μg/mouse), or PBS control in the presence of aluminum adjuvants (500 μg/mouse, InvivoGen) via intramuscular route. The immunized mice were boosted 14 days later with the same dose immunogen and adjuvants, and sera were collected at 14, 21 and 42 days after the 2nd immunizations for specific IgG antibodies or neutralizing antibodies analysis.

**ELISA**

ELISA was carried out to detect the binding of Fc-RBD_Omicron_ to soluble hACE2 protein, and human IgG Fc (Fc, Thermo Fisher Scientific) protein were used as control. Briefly, ELISA plates were precoated with SARS-CoV-2 RBD or Fc protein (1 μg/ml) overnight at 4 °C and blocked with 2% fat-free milk in PBST for 2 h at 37 °C. Serially diluted His_×6_ tagged hACE2 protein (Sino Biological) was added to the plates and incubated for 2 h at 37 °C. After four washes, the bound protein was detected using anti-His tag antibody (HRP) (0.005 μg/ml, Sino Biological) for 1 h at room temperature. The reaction was visualized by addition of substrate 3,3’,5,5’-Tetramethylbenzidine (TMB, Sigma) and stopped by H_2_SO_4_ (1 N). The absorbance at 450 nm was measured by an ELISA plate reader.

Next, ELISA was conducted to detect the binding of SARS-CoV-2 Fc-RBD_Omicron_ protein to FNP, and ovalbumin (OVA, Invivogen) was set as controls. Like the above description, ELISA plates were precoated with Fc-RBD_Omicron_ or OVA at 1 μg/ml. And then His_×8_ tagged FNP protein (0.5 μg/ml) was added to the wells and incubated for 2 h at 37 °C. After four washes, the binding was detected using HRP labeled anti-His tag antibody (0.005 μg/ml, Sino Biological) for 1 h at room temperature. The reaction was visualized by addition of TMB (Sigma) and stopped by H_2_SO_4_ (1 N). The absorbance at 450 nm was measured by an ELISA plate reader.

ELISA was also performed to detect the interaction between SARS-CoV-2 Omicron RBD protein and RBD-specific antibodies in mouse sera. The procedure was the same as described above, except that the ELISA plates were coated with RBD (Sino Biological) at 1 μg/ml and then sequentially incubated with serially diluted mouse sera and HRP-conjugated anti-mouse antibodies (1:5000, Thermo Fisher Scientific).

**Live SARS-CoV-2 neutralization assay**

A neutralization assay of live SARS-CoV-2 was performed using a cytopathic effect (CPE) assay in a biosafety level 3 laboratory. Briefly, heat inactivated sera samples were serially diluted starting at 1:20. Triplicates of each mAb dilution were incubated with the same volume of 100 TCID_50_ of the SARS-CoV-2 Omicron variant (clinical isolate) incubated at 37℃ for 1 h. Mixtures were then transferred to 96-well plates containing Vero cells. After incubation at 37°C for 3 days. CPEs were confirmed for each well in a blinded fashion by two independent observers. The neutralization titer (NT_50_) was calculated as the highest sample dilution that protected 50% of the wells.

**Pseudovirus neutralization and inhibition assays**

SARS-CoV-2 pseudovirus was generated, as previously described.^2^ Briefly, HEK293T cells were cotransfected with a plasmid encoding Env-defective, luciferase-expressing HIV-1 genome (pNL4-3.luc.RE) and a plasmid encoding SARS-CoV-2 S protein using the calcium phosphate method. The transfected medium was replaced by fresh DMEM 8 h later, and pseudovirus-containing supernatants were collected 48 h later for single-cycle infection in hACE2/HEK293T cells. Pseudovirus neutralization assay was then performed by incubation of SARS-CoV-2 pseudovirus with serially diluted mouse sera for 1 h at 37 °C, followed by addition of the mixture into hACE2/HEK293T cells. Fresh medium was added 24 h later, and the cells were lysed 72 h later in cell lysis buffer (Promega). The lysed cell supernatants were incubated with luciferase substrate (Promega) and detected for relative luciferase activity. The 50% pseudovirus neutralizing antibody titer (NT_50_) was calculated.

**Flow cytometry**

Flow cytometry analysis was first performed to detect the binding of the Fc-RBD_Omicron_ protein to hACE2/HEK293T cell.^3^ The Fc protein was used as controls. Briefly, cells were incubated with reciprocal diluted Fc-RBD_Omicron_ for 30 min at room temperature, after three washes with PBS (containing 2% FBS), the cells were incubated with FITC-labeled goat anti-human IgG antibody (1:500, Thermo Fisher Scientific) at room temperature for 20 min. After washes, the cells were fixed with 4% formaldehyde and the fluorescence intensity of the cells was measured using flow cytometry (BD LSRFortessa^TM^ system).

Flow cytometry analysis was next conducted to detect the interaction between the Fc-RBD_Omicron_ and hACE2 in the presence of mouse sera. Briefly, hACE2/HEK293T cells were incubated with Fc-RBD_Omicron_ (10 μg/ml) in the presence or absence of serially diluted mouse sera at room temperature for 1 h, which was followed by incubation with FITC-labeled goat anti-human IgG antibody (1:500, Thermo Fisher Scientific) for 30 min and analyzed.

**ELISpot assay**

Splenocytes collected from immunized mice were stimulated with RBD proteins (Sino Biological) at a concentration of 5 μg/ml for 24 h. Antigen specific cells were detected by mouse IFN-γ and IL-4 ELISpot kits (Abcam) according to the manufacturer’s protocols. The unstimulated cells were used as controls. Antigen-specific spots were then counted using an S6 ultra immunoscan reader (Cellular Technology Ltd.), and the number of IFN-γ- or IL-4-positive T cells was analyzed by ELISpot Reader (AID GmbH). The results were showed as the number of spots per million splenocytes.

**Statistical analysis**

The results are presented as the mean±standard error of mean (S.E.M.). The difference between any two groups were determined by unpaired parametric *t*-test or one-way ANOVA with multiple comparisons tests depending on the distribution of the data. Power analysis was conducted to confirm that the powers of the tests used were above 80%, which are the probabilities of accepting true hypotheses and making true-positive detection of the treatment effects.^4,5^ All data were analyzed with GraphPad Prism version 8.0 software.

Figure. S1

**
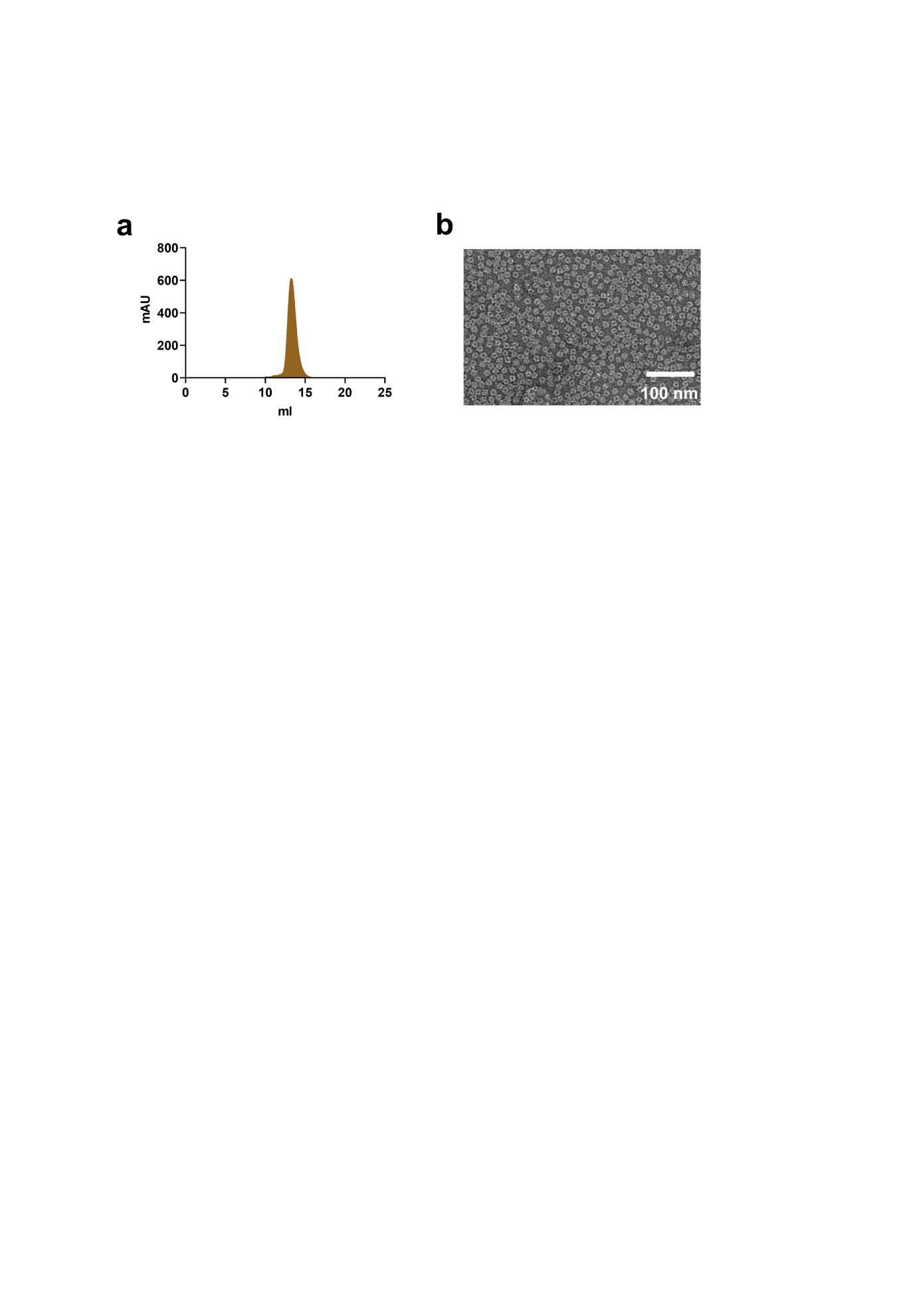
**

**Supplementary Fig. S1. Preparation and characterization of 24-meric ferritin-based nanoparticle.**

The ferritin with an N-terminal protein A tag was expressed in BL21 (DE3) *E. coli* cells, and then purified to high quality. **a** Representative elution profiles of the nanoparticles from Superdex 200 Increase 10/300 GL gel filtration chromatography recorded at milli-absorbance unit (mAu) at 280 nm wavelength. **b** Negative-staining EM analysis of the nanoparticles. Scale bar, 100 µm.

Figure. S2


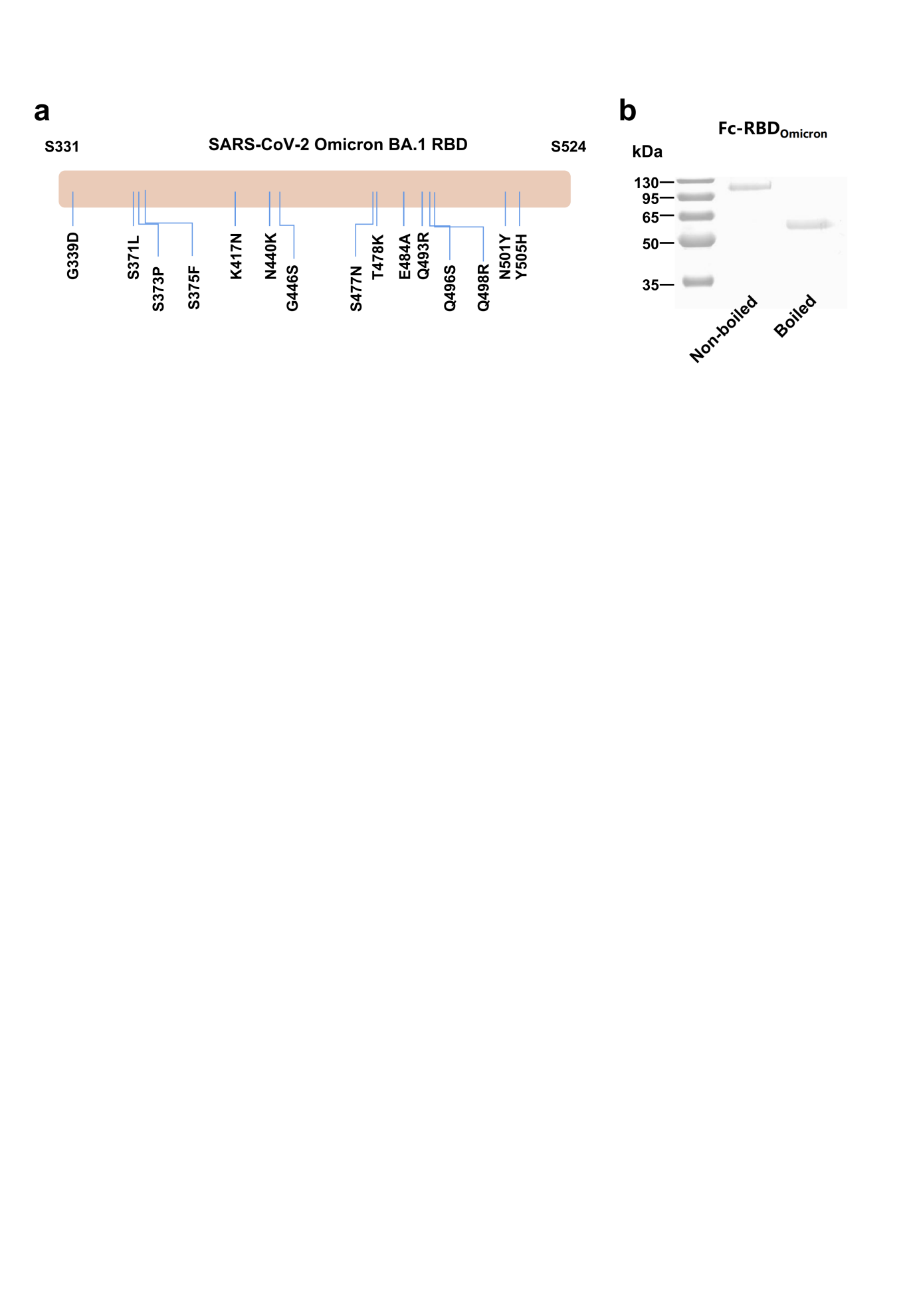


**Supplementary Fig. S2. Characterization of the Fc tagged RBD of SARS-CoV-2 Omicron variant.**

**a** The schematic diagram showing the RBD (residues 331aa-524aa of the spike) mutations of the SARS-CoV-2 Omicron variant. Compared to the Wildtype strain, total 15 mutations were included and labeled. **b** Expression of Fc-RBD_Omicron_ protein in the FreeStyle 293-F cells. Cells were transfected with Omicron RBD-encoding plasmid, and the supernatant was purified with protein A beads and gel filtration chromatography at 72 h after transfection. and then the boiled or non-boiled Fc-RBD_Omicorn_ samples was analyzed by SDS-PAGE under reducing condition, the molecular weight markers were indicated on the left.

Figure. S3

**
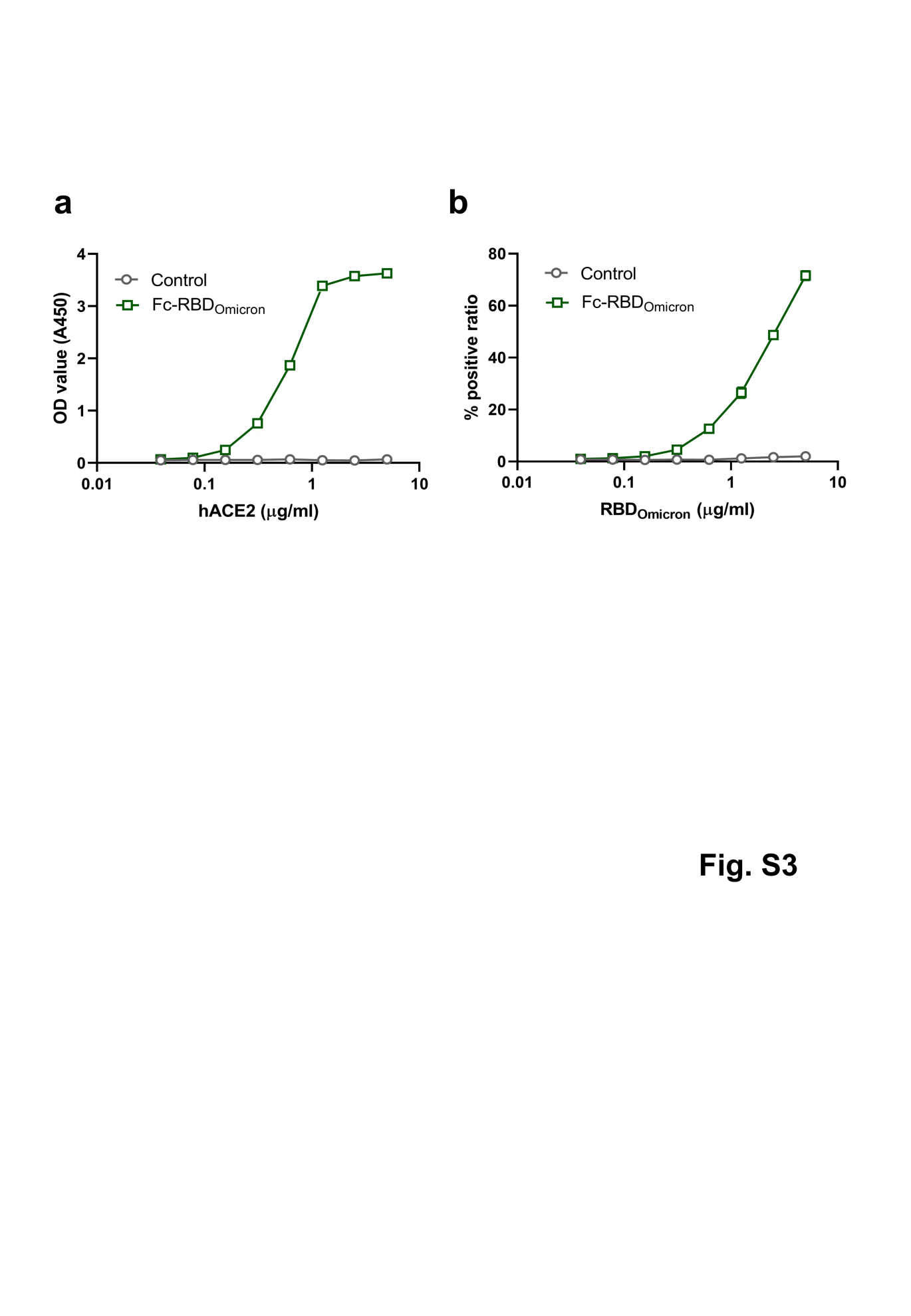
**

**Supplementary Fig. S3. The Fc-RBD_Omicron_ showed high potency of receptor binding ability.**

**a** Detection of Fc-RBD_Omicron_ binding to hACE2 by ELISA. The data are presented as mean±S.E.M. (n=3). **b** Binding of Fc-RBD_Omicron_ protein to hACE2/HEK293T cells by flow cytometry. The data are presented as mean±S.E.M. (n=3). Human IgG Fc protein acted as a control. Experiments were repeated twice with similar results.

Figure. S4


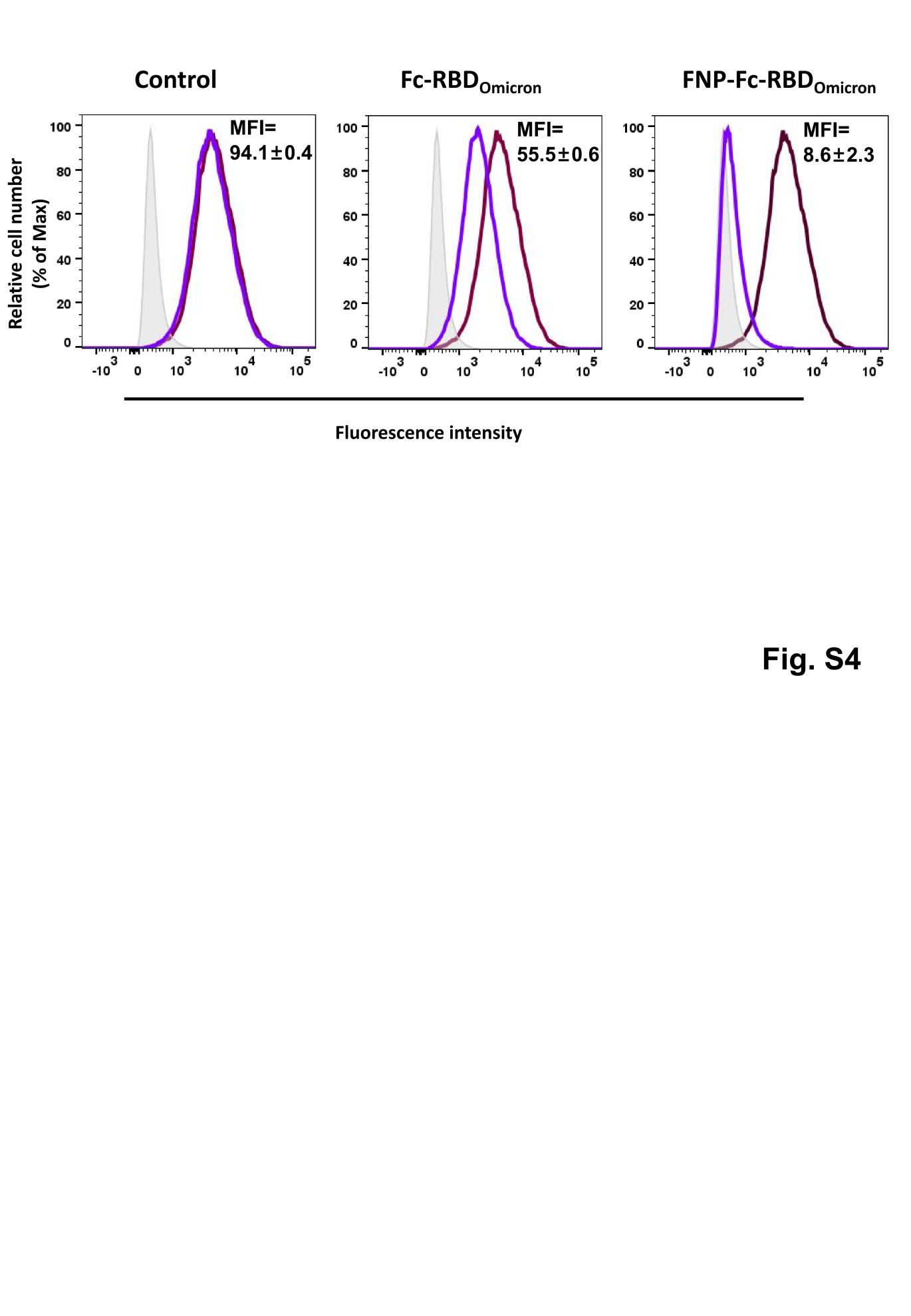


**Supplementary Fig. S4.** **Neutralizing mechanism analysis of vaccine-induced antibodies.**

Representative images of the RBD-receptor binding inhibition by the sera (1:40) from mice immunized with PBS (left panel), Fc-RBD_Omicron_ (middle panel), or FNP-Fc-RBD_Omicron_ (right panel). The violet lines represented median fluorescence intensity (MFI) values. The binding between Fc-RBD_Omicron_ and hACE2 is shown in dark red lines. Light gray shades indicate the Fc-hACE2 binding.

Figure. S5


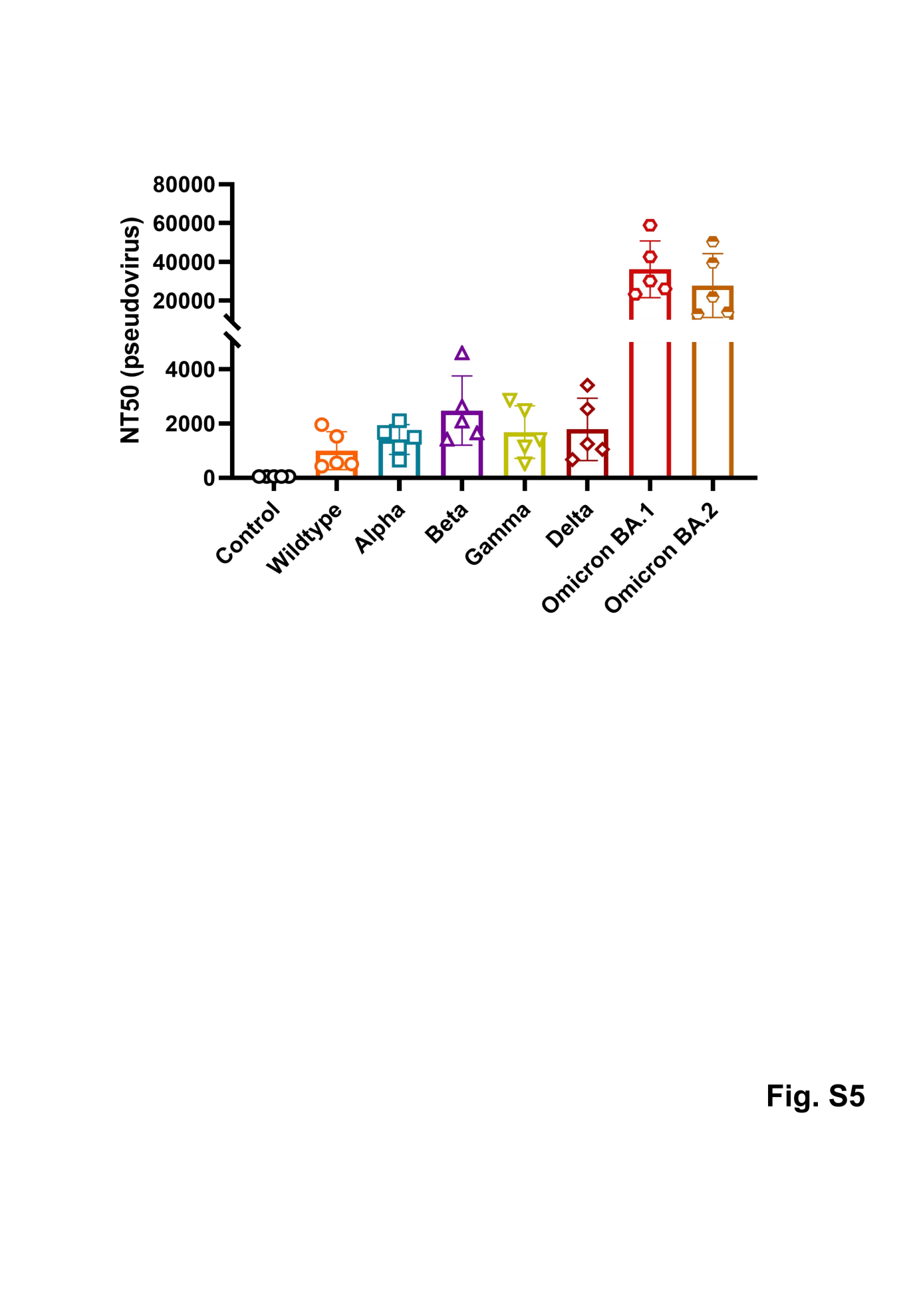


**Supplementary Fig. S5. Antibodies induced by the FNP-Fc-RBD_Omicron_ immunization neutralize pseudotyped SARS-CoV-2 variants.**

The cross-neutralizing antibodies from FNP-Fc-RBD_Omicron_ immunized sera (3-fold serial dilutions from 1:50) were assessed to interrupt the cellular entry of pseudoviruses of SARS-CoV-2 VOCs (Wildtype, Alpha, Beta, Gamma, Delta, Omicorn BA.1, and Omicron BA.2) in hACE2/HEK293T cells. The data are presented as mean±S.E.M. (n=5). Experiments were repeated twice with similar results.

Figure. S6


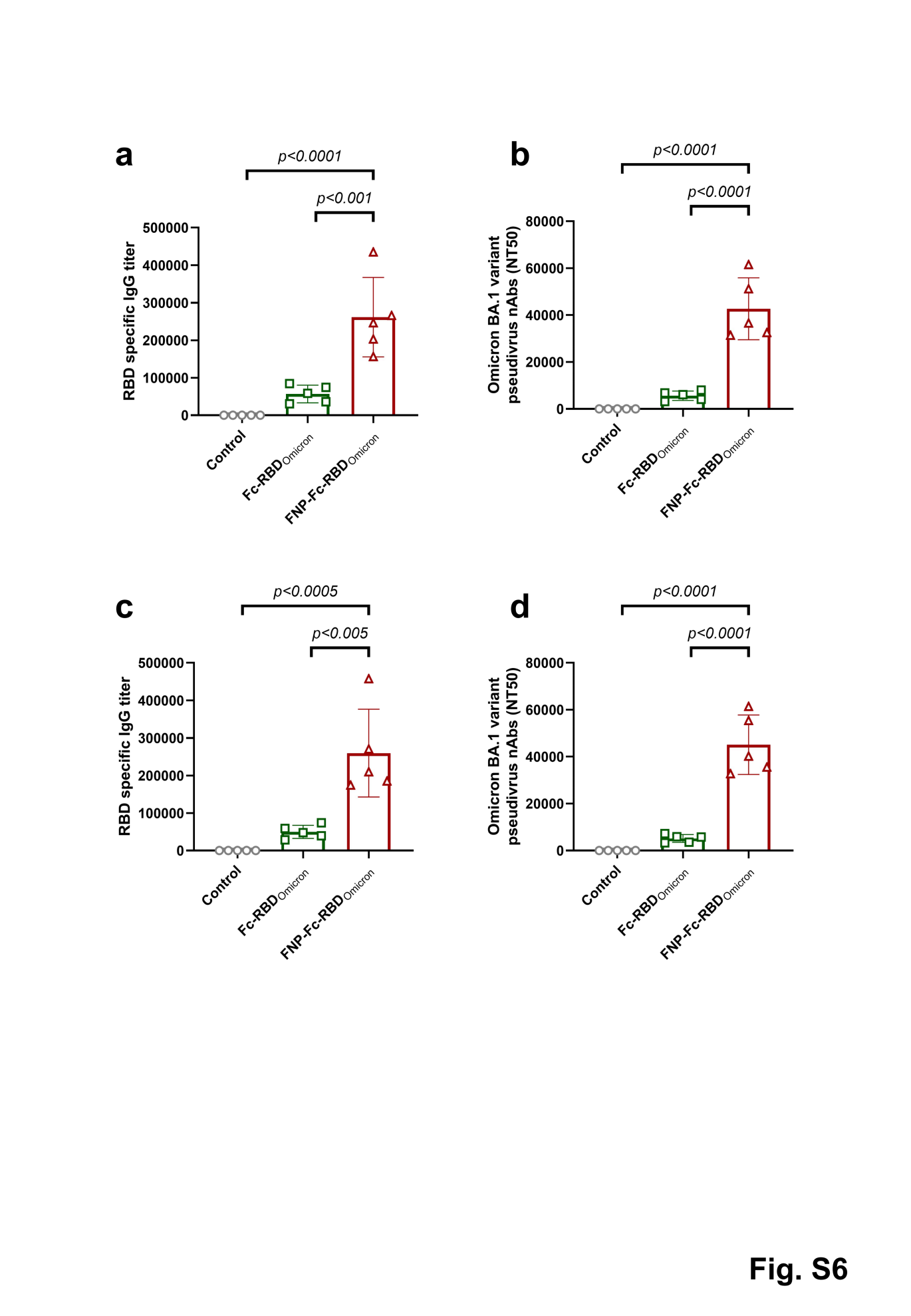


**Supplementary Fig. S6. Prolonged specific and neutralizing antibodies induced by the FNP-Fc-RBD_Omicron_.**

The mice were immunized via intramuscular (i.m.) prime and boost at 2 weeks (10 μg per mouse, n=5). **a, c** Sera at 21 (**a**) and 42 (**c**) days post-2nd immunization were detected for RBD_Omicron_-specific IgG antibodies by ELISA. **b, d** The neutralizing antibodies at 21 (**b**) and 42 (**d**) days post-2nd immunization were assessed by pseudotyped SARS-CoV-2 Omicron BA.1 virus. The data are presented as mean±S.E.M. (n=5). Statistical significance was calculated via one-way ANOVA with multiple comparisons test. Experiments were repeated twice with similar results.

Figure. S7


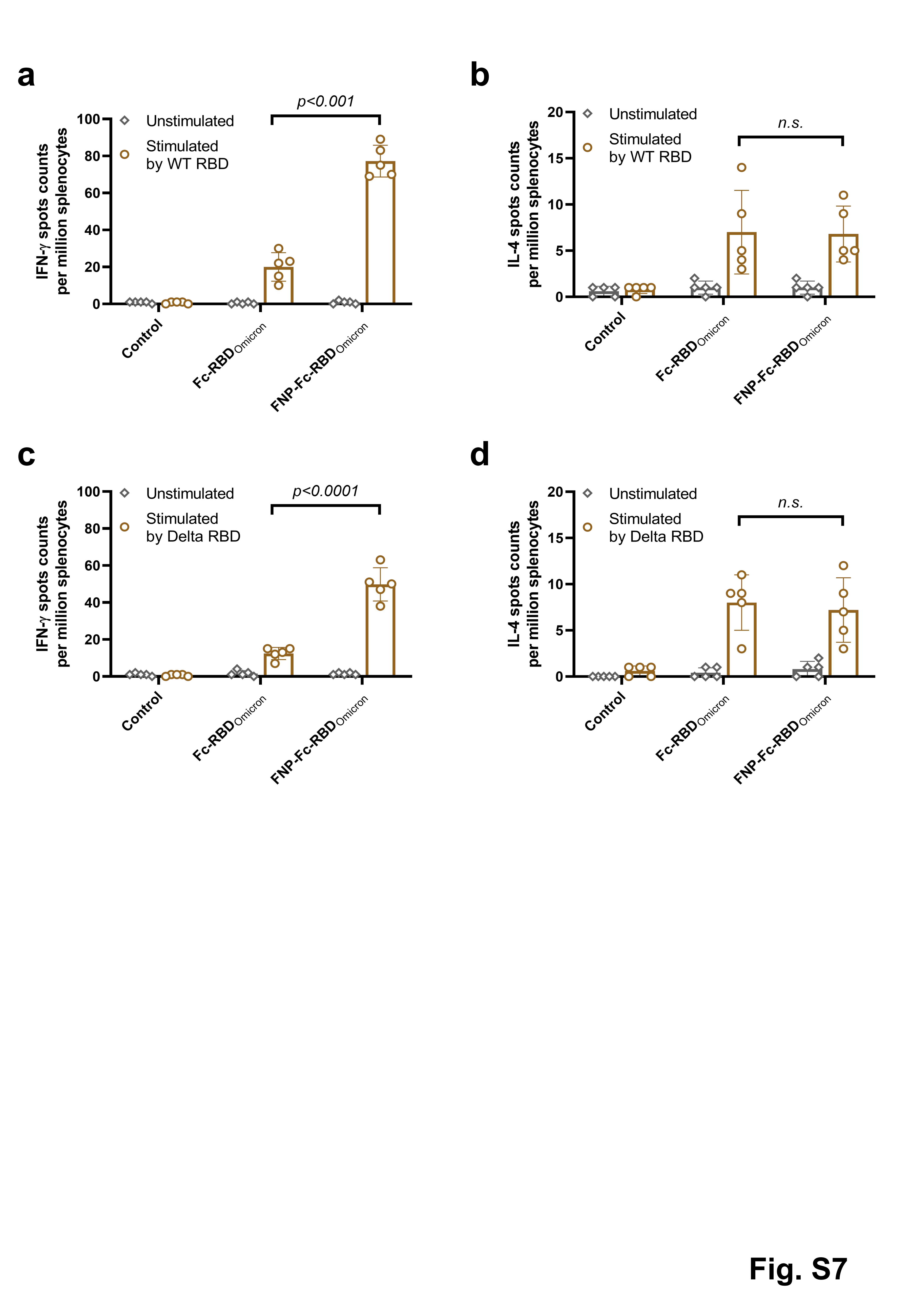


**Supplementary Fig. S7. A Th1 biased cellular immune response induced by FNP-Fc-RBD_Omicron_.**

Splenocytes were stimulated with RBD proteins of Wildtype (WT) (**a, b**) and Delta (**c, d**) strains. The IFN-γ and IL-4 secretion condition in splenocytes were detected by ELISpot assays. Data represented as mean±S.E.M. (n = 5). Statistical significance was calculated via unpaired parametric *t*-test.

**References**

1. Wan, Y, *et al*. Molecular mechanism for antibody-dependent enhancement of coronavirus entry. *J. Virol.* **94** (2020).

2. Tai, W, *et al*. Identification of SARS-CoV RBD-targeting monoclonal antibodies with cross-reactive or neutralizing activity against SARS-CoV-2*. Antiviral Res.* **179**,104820 (2020).

3. Tai, W, *et al*. A novel receptor-binding domain (RBD)-based mRNA vaccine against SARS-CoV-2*.* *Cell* **30**, 932-935 (2020).

4. Wittes, J. Sample size calculations for randomized controlled trials. *Epidemiol. Rev.* **24**, 39-53 (2002).

5. Lenth, R. Some practical guidelines for effective sample size determination. *Am. Stat.* **55**, 187-193 (2001).
